# Hydrolysis of the Rutinose-Conjugates Flavonoids Rutin and Hesperidin by the Gut Microbiota and Bifidobacteria

**DOI:** 10.3390/nu7042788

**Published:** 2015-04-14

**Authors:** Alberto Amaretti, Stefano Raimondi, Alan Leonardi, Andrea Quartieri, Maddalena Rossi

**Affiliations:** Department of Life Sciences, University of Modena and Reggio Emilia, via Campi 183, 41125 Modena, Italy; E-Mails: alberto.amaretti@unimore.it (A.A.); stefano.raimondi@unimore.it (S.R.); alan.leonardi@unimore.it (A.L.); andrea.quartieri@unimore.it (A.Q.)

**Keywords:** *bifidobacterium*, hesperidin, hesperetin, rutin, quercetin, rutinosides, polyphenols

## Abstract

Flavonols and flavanones are polyphenols exerting many healthy biological activities. They are often glycosylated by rutinose, which hampers absorption in the small intestine. Therefore they require the gut microbiota to release the aglycone and enable colonic absorption. The role of the gut microbiota and bifidobacteria in the release of the aglycones from two major rutinosides, hesperidin and rutin, was investigated. In bioconversion experiments, the microbiota removed rutinose from both rutin and hesperidin, even though complete hydrolysis was not obtained. To investigate whether bifidobacteria can participate to the hydrolysis of rutinosides, 33 strains were screened. Rutin was resistant to hydrolysis by all the strains. Among six tested species, mostly *Bifidobacterium catenulatum* and *Bifidobacterium pseudocatenultum* were able to hydrolyze hesperidin, by means of a cell-associated activity. This result is in agreement with the presence of a putative α-l-rhamnosidase in the genome of *B. pseudocatenulatum*, while most of the available genome sequences of bifidobacteria aside from this species do not bear this sequence. Even though *B. pseudocatenulatum* may contribute to the release of the aglycone from certain rutinose-conjugated polyphenols, such as hesperidin, it remains to be clarified whether this species may exert a role in affecting the bioavailability of the rutinoside *in vivo*.

## 1. Introduction

Plants are dietary sources of hundreds of polyphenols with beneficial biological activities. Polyphenols are classified as phenolic acids, flavonoids, stilbenes, lignans, hydrolyzable tannins, and condensed tannins on the basis of the number of phenol rings and the structure elements which connect them, with flavonoids clustered into flavonols, flavones, isoflavones, flavanones, anthocyanidins, and flavanols [1]. Likewise other flavonoids, flavonols and flavanones are antioxidants, and exert many health effects which include anti-inflammatory effects and protective activities against breast and gastrointestinal cancer and cardiovascular diseases [2,3,4].

**Figure 1:**
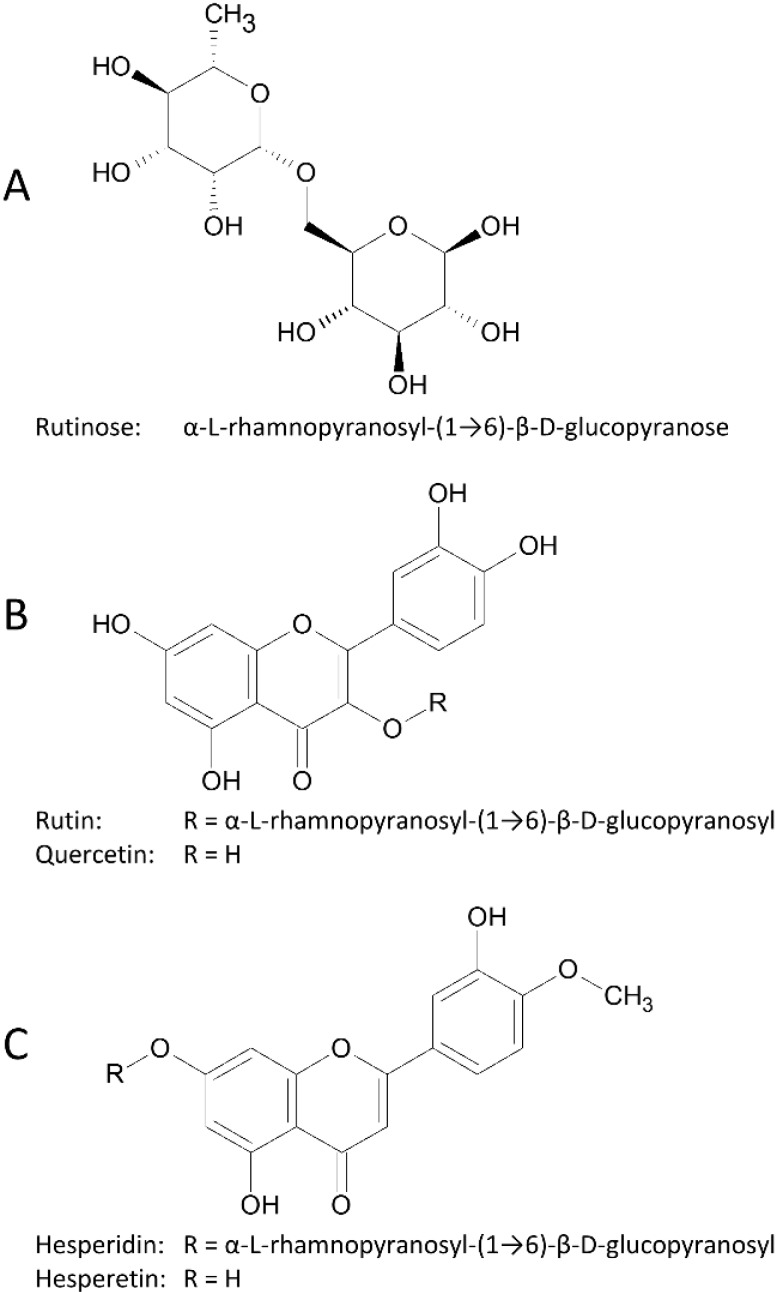
Structure of rutinose (**A**); rutin and quercetin (**B**); hesperidin and hesperetin (**C**).

Flavonols are the most ubiquitous flavonoids in food, particularly in onions, vegetables, berries, wine, and tea. Quercetin is the most abundant flavonol, followed by kaempferol. These compounds generally occur in glycosylated form, bound to glucose, rhamnose, or rutinose, the most widespread glycosidic form of quercetin being its rutinoside, rutin (Figure 1) [1]. Flavanones are less common than flavonols, but they are present in particularly high concentrations in citrus fruit, particularly in the albedo. Hesperetin, naringenin, and eriodictyol are the most common flavanones. They are generally glycosylated by disaccharides, such as rutinose or neohesperidose. The most widespread glycosidic form of hesperetin in citrus is the rutinoside, hesperidin (Figure 1) [1].

The sugar moiety has a major impact on the absorption of flavonols and flavanones in the small intestine [5]. Conjugation with glucose enhances quercetin absorption from the small intestine, through mechanisms that certainly involve active transport of the glucoside. On the other hand, the other glyco-conjugates of quercetin are poorly absorbed in the upper tract of the intestine and reach the hindgut where they encounter the resident microbiota. In the colon, bacteria could hydrolyze rutin or other glyco-conjugates, thus removing the sugar moiety and enabling the absorption of the aglycone [6,7,8,9,10]. As with flavonols, the conjugation to rutinose limits the absorption of flavanones in the small intestine. Therefore, the rutinosides of flavanones also require hydrolysis by colonic bacteria to release the aglycone, and to enable absorption in the colon [11,12].

Among indigenous bacteria of human gut microbiota, the genus *Bifidobacterium* is the most relevant health-promoting group [13]. Bifidobacteria are obligate saccharolytic fermentative bacteria which exert beneficial health effects through a variety of different mechanisms [14,15,16]. It was also hypothesized that they may be involved in the release of aglycones from glyco-conjugated forms of polyphenols [17]. Having adapted within an ecosystem where indigestible oligo- and polysacchardies are the major fermentable carbon source, bifidobacteria bear a number of glycosyl hydrolases that may potentially be involved also in the hydrolysis of glyco-conjugate forms of phytochemicals [18,19]. As a matter of fact, some species of *Bifidobacterium* can hydrolyze the soy isoflavones daidzin, the lignan secoisolariciresinol-diglucoside, and chlorogenic acid [20,21,22,23]. This study aims to focus on the involvement of bifidobacteria in the hydrolysis of the major rutinosides rutin and hesperidin, taking into account the inter-individual variability of transformation of these conjugated flavonoids by colonic microbiota.

## 2. Experimental Section

### 2.1. Chemicals and Bacterial Strains

All chemicals were purchased from Sigma-Aldrich (Steinheim, Germany) unless otherwise stated. Hesperetin, hesperidin, quercetin, and rutin were dissolved at the concentration of 250 mM in DMSO. The stock solutions were diluted in DMSO to prepare the analytical standards. Appropriate volumes were added to bioconversion mixtures to achieve 500 μM, this concentration being within the range utilized in previous studies with other phytochemicals and consistent with reasonable dietary intake [1,21,24].

Thirty-three intestinal strains of bifidobacteria were taken from our culture collection or obtained from the ATCC (Table 1). All the strains were anaerobically cultured at 37 °C in Lactobacilli MRS broth (BD Difco, Sparks, NV, USA) supplemented with 0.5 g/L l-cysteine HCl, hereinafter referred to as MRS. Growth was monitored by measuring the turbidity at 600 nm (OD_600_).

**Table 1:** Hydrolysis of rutin and hesperidin by cultures of *Bifidobacterium* strains.

Strain	Rutin → Quercetin	Hesperidin → Hesperetin
	Bioconversion %	Bioconversion %
*B. animalis* subsp. *animalis* ATCC 27536	-	-
*B. animalis* subsp. *animalis* WC 0409	-	-
*B. animalis* subsp. *animalis* WC 0410	-	-
*B. animalis* subsp. *animalis* WC 0411	-	-
*B. animalis* subsp. *lactis* WC 0412	-	-
*B. animalis* subsp. *lactis* WC 0413	-	-
*B. animalis* subsp. *lactis* WC 0414	-	-
*B. animalis* subsp. *lactis* WC 0432	-	-
*B. animalis* subsp. *lactis* WC 0471	-	-
*B. bifidum* WC 0415	-	-
*B. bifidum* WC 0417	-	-
*B. bifidum* WC 0418	-	-
*B. breve* WC 0420	-	-
*B. breve* WC 0421	-	-
*B. breve* WC 0422	-	4a
*B. breve* WC 0423	-	-
*B. breve* WC 0424	-	-
*B. breve* WC 0473	-	-
*B. catenulatum* ATCC 27539	-	6a
*B. longum* subsp. *infantis* ATCC 15697	-	-
*B. longum* subsp. *infantis* WC 0433	-	-
*B. longum* subsp. *infantis* WC 0434	-	-
*B. longum* subsp. *longum* WC 0436	-	-
*B. longum* subsp. *longum* WC 0438	-	-
*B. longum* subsp. *longum* WC 0439	-	-
*B. longum* subsp. *longum* WC 0440	-	-
*B. longum* subsp. *longum* WC 0443	-	-
*B. pseudocatenulatum* WC 0400	-	9a
*B. pseudocatenulatum* WC 0401	-	6a
*B. pseudocatenulatum* WC 0402	-	-
*B. pseudocatenulatum* WC 0403	-	46c
*B. pseudocatenulatum* WC 0407	-	5a
*B. pseudocatenulatum* WC 0408	-	16b

Bifidobacteria were cultured for 48 h in MRS supplemented with 500 μM rutinoside. Values are means, *n* = 3. Within a column, means with different letters significantly differ (*p* < 0.05). “-” indicates the absence of detectable hydrolysis products.

### 2.2. Bioconversion of Hesperidin and Rutin by Fecal Bacteria

The fecal specimens that were utilized for bioconversion experiments were collected after obtaining written informed consent from nine healthy volunteers (six men and three women, aged 25 to 50) who had not been treated with prebiotics and/or probiotics for one month, and antibiotics for at least three months. The subjects joined the study voluntarily after being informed of the study (aim, experimental scheme, and expected results), and that they would not be subjected to any treatment, and that personal data and specimens would be confidential.

Feces were suspended 10% w/v in phosphate buffered saline, containing 0.5 g/L l-cysteine HCl (pH adjusted to 6.5), hereinafter referred to as PBS-Cys. To perform experiments with reproducible inocula, suspensions were supplemented with 100 g/L glycerol and stored at −20 °C until use. Preparation was performed in an anaerobic cabinet (Anaerobic System, Forma Scientific, Marietta, OH, USA) under an 85% N_2_, 10% CO_2_, 5% H_2_ atmosphere.

For bioconversion experiments, 1 mL of fecal suspension was anaerobically diluted with 4 mL of PBS-Cys, and supplemented with 500 μM hesperidin or rutin. The samples were anaerobically incubated at 37 °C for 24 h and analyzed for bioconversion yield. Blank controls containing the same nine fecal samples incubated for 24 h in PBS-Cys were prepared. As control, 500 μM hesperidin or rutin was incubated in PBS buffer for 24 h.

### 2.3. Bioconversion of Hesperidin and Rutin by Bifidobacteria

Overnight cultures of bifidobacteria were inoculated (10% v/v) into 5 mL of MRS supplemented with 500 μM hesperidin or 500 μM rutin. After 48 h of anaerobic incubation at 37 °C under gentle agitation, cultures were analyzed for rutinosides and aglycones concentrations. As controls, cultures without rutinosides and non-inoculated MRS containing 500 μM hesperidin or 500 μM rutin were analyzed after 48 h of anaerobic incubation at 37 °C.

Further bioconversion experiments were performed with supernatants and cell extracts from *Bifidobacterium* MRS cultures. Supernatant and biomass were separated by centrifugation (6000× *g* for 10 min at 4 °C). The pH of the supernatant was corrected to 6.5, then sterilized through a 0.22 μm filter. The biomass was washed twice and resuspended with PBS-Cys, in order to obtain the turbidity of 1.5 AU (λ = 600 nm), and subjected to mechanical disruption through one passage at 20.0 kPsi in One Shot Cell Disrupter (Constant Systems). The cell extract was clarified by centrifugation (13,000× *g* for 15 min at 4 °C), and liquid phase was sterilized through a 0.22 μm filter.

Supernatants and cell-free extracts were supplemented with 500 μM hesperidin or rutin and incubated anaerobically at 37 °C under gentle agitation. After 24 h of incubation, the samples were heated at 100 °C for 10 min to stop enzymatic reactions, and analyzed. As controls, supernatants or cell extracts without rutinosides, or 500 μM rutinosides in MRS or PBS-Cys were utilized.

### 2.4. Fermentation Experiments

Bioreactor batch processes were performed with *B. pseudocatenulatum* WC 0403 in 500 mL bioreactors (Sixfors V3.01, Infors, Bottmingen, Switzerland), containing 250 mL of MRS supplemented with 500 μM hesperidin. The culture was kept at 37 °C under gentle agitation; anaerobic conditions were maintained by keeping the medium under a stream of CO_2_. A pH controller delivered 4 M NaOH to prevent the pH falling below 5.5. Samples were collected periodically to measure glucose, hesperetin, and hesperidin.

To study hesperidin bioconversion by the supernatant and cell extracts at different growth phases, samples were withdrawn after 8, 24, and 48 h from controlled-pH (5.5) batch cultures.

### 2.5. Analysis of Flavonols and Flavanones

At the end of the transformation experiments carried out with cultures, supernatants, and cell extracts, the bioconversion mixtures were frozen at −80 °C, freeze dried, and extracted with DMSO. The extracts were clarified by centrifugation (13,000×g for 5 min at 4 °C) and filtration (0.22 μm) to remove cells and/or solids, and analyzed with HPLC to quantify residual rutinosides and the corresponding aglycones. One μL was injected into a HPLC device (Agilent 1100, Agilent Technologies Inc., Santa Clara, CA, USA) equipped with a ZORBAX Eclipse XDB-C18 column (rapid resolution, 1.8 μm particle size, 4.6 × 50 mm, Agilent,) and variable wavelength detector coupled with an ESI ion trap mass spectrometer (Agilent 1100 LC-MSD trap). The mobile phase was composed of 0.1% (v/v) acetic acid in water (solvent A) and 0.1% acetic acid in acetonitrile (solvent B). The flow rate was 0.7 mL/min. The following gradient of solvent B was applied: 0–25 min, linear from 10% to 30%; 25–30 min, linear to 50%; 30–40 min, linear to 100%; 40–47 min, linear to 10%. Rutin, hesperidin, quercetin, and hesperetin were recognized based on the retention time (13.5, 17.6, 23.3, and 29.3 min respectively) and the *m/z* of the parent ion. The analytes were quantified at 280 nm using the external standard method, the linearity being demonstrated from 10 to 1000 μM (*R*^2^ > 0.997). The limits of detection (LOD) of rutin, hesperidin, quercetin, and hesperetin were 27, 21, 61, and 43 μM, respectively, calculated as 3·(Sy/x)/b, where Sy/x represents the residual standard deviation and b is the slope of the linear calibration.

### 2.6. Statistical Analysis

All values are means of three independent experiments. Means were compared with Student’s *t*-test. Differences were considered statistically significant for *p* < 0.05.

## 3. Results

### 3.1. Bioconversion of Rutin and Hesperidin by Resting Cells of Human Gut Microbiota

**Figure 2:**
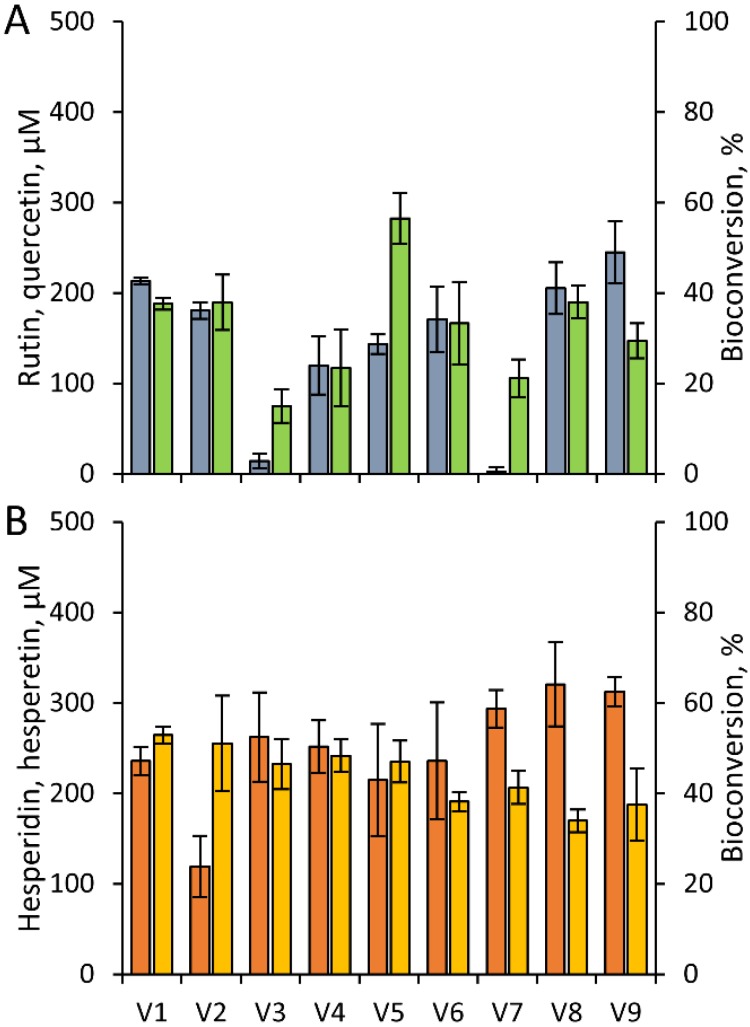
Bioconversion of rutin (**A**) and hesperidin (**B**) with cells of human gut microbiota. Fecal bacteria from nine subjects were suspended in PBS-Cys and incubated anaerobically at 37 °C for 24 h in presence of 500 μM rutin or hesperidin. Legend: rutin, grey; quercetin, green; hesperidin, orange; hesperetin, yellow. Values are means ± SD, *n* = 3.

The transformation of rutin and hesperidin was observed after 24 h incubation with the resting cells of human gut microbiota from nine different healthy volunteers. All the fecal suspensions succeeded in releasing quercetin from rutin, with yields ranging from 15% to 38% (Figure 2A). After the incubation, the sum of residual rutin and quercetin on molar basis was generally significantly lower than the initial rutin concentration, ranging from 18% to 85%. In subjects V3 and V7, rutin got nearly exhausted while quercetin did not account for more than 21%, indicating that further biotransformations, other than rutinose removal, occurred. Similarly, all the fecal suspensions succeeded in releasing hesperetin, with yields ranging from 34% to 53% (Figure 2B). In this case, the recovery of hesperetin and hesperidin was high, above the 90% in most of the samples.

No relationship could be established between the extent of the hydrolysis of rutin and hesperidin (*R^2^* < 0.01).

### 3.2. Bioconversion of Rutin and Hesperidin by Bifidobacteria

The ability of bifidobacteria to hydrolyze rutin and hesperidin was investigated on thirty-three human strains of *Bifidobacterium*, belonging to the species *B. animalis* subsp. *animalis*, *B. animalis* subsp. *lactis*, *B. bifidum*, *B. breve*, *B. catenulatum*, *B. longum* subsp. *infantis*, *B. longum* subsp. *longum*, and *B. pseudocatenulatum*. They all were incapable to transform rutin during 48 h of growth in rutin-supplemented MRS (Table 1). Consistently, both the supernatants and the cell extracts of all the strains were also inactive towards rutin. Furthermore, when bifidobacteria were cultured in presence of quercetin, they all failed to transform this aglycone.

Unlike rutin, a few strains were capable of hydrolyzing hesperidin to some extent, yielding hesperetin (Table 1). This feature was a property of the strains belonging to the species *B. catenulatum* and *B. pseudocatenulatum* (mean conversion = 12.6%, median = 6%), *B. pseudocatenulatum* WC 0403 yielding the highest amount of hesperetin (46% conversion). The strains belonging to the other species were unable to release the aglycone, with the exception of *B. breve* WC 0422. In all the strains, the sum of the aglycone, if any, and the residual rutinoside was close to 500 μM. Consistently, when bifidobacteria were cultured for 48 h in hesperetin-supplemented medium, the aglycone remained unaltered. The supernatants of all the tested bifidobacteria were inactive in hesperidin hydrolysis. Cell extracts of the strains whose culture was active released hesperetin from hesperidin, whereas those of the inactive strains did not.

### 3.3. Kinetics of Hesperidin Hydrolysis by B. Pseudocatenulatum WC 0403

**Figure 3:**
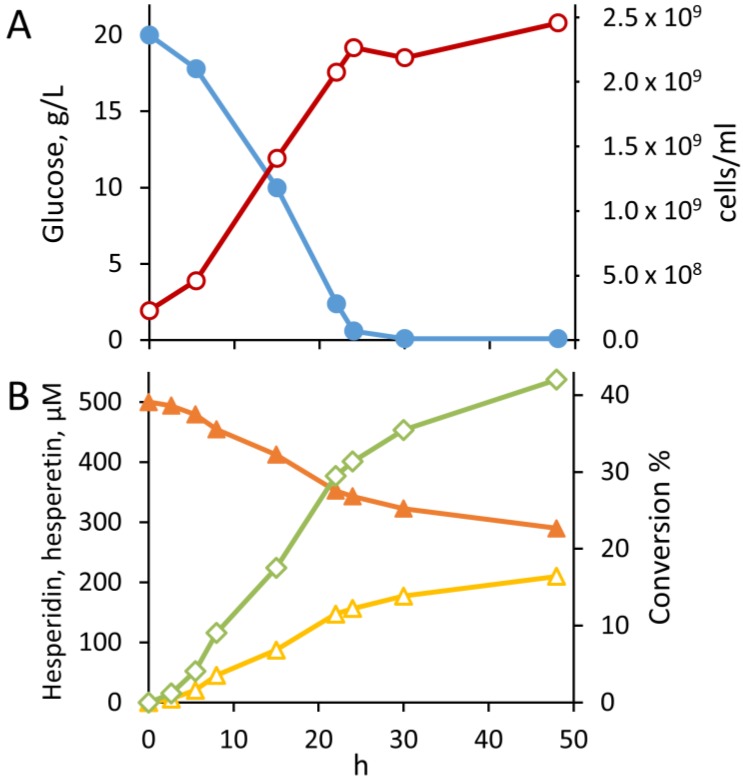
Timecourse of hesperidin hydrolysis in batch cultures of *B. pseudocatenulatum* WC 0403. Legend: cell counts, ○; glucose, ●; hesperidin, ▲; hesperetin, ∆; conversion, ◊. The experiment was carried out in triplicate. Data from one of the repetitions are reported herein.

*B. pseudocatenulatum* WC 0403, which exhibited the highest yield of hesperidin hydrolysis, was selected for a deeper investigation in bioreactor batch cultures of the transformation kinetics of hesperidin. The hydrolysis occurred especially during the growth phase, *i.e.*, during the first 24 h of cultivation, when 31% conversion was achieved. During the stationary phase, a minor hesperidin conversion was observed, with a 42% final yield after 48 h (Figure 3).

**Figure 4:**
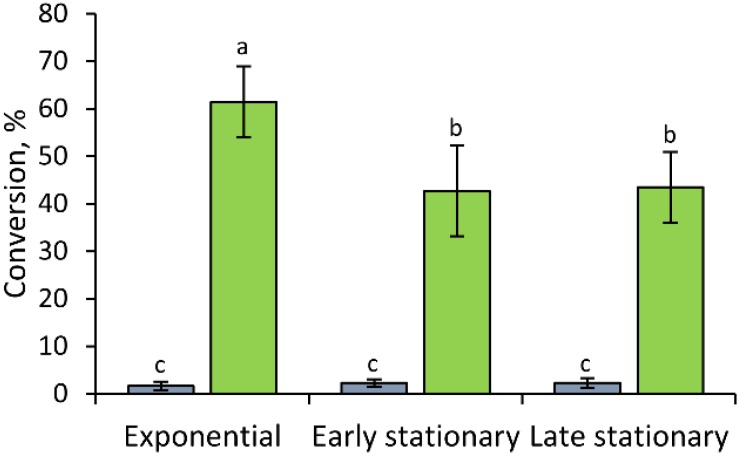
Hesperetin production after 24 h incubation of 500 μM hesperidin with the supernatants (grey) and the cellular extracts (green) of *B. pseudocatenulatum* WC 0403. Cellular extracts were preliminary normalized to the same cellular concentration. Values are means ± SD, *n* = 3. Within each series, means without a common letter significantly differ (*p* < 0.05).

In order to investigate the hydrolytic activity against hesperidin during the different growth phases, samples of MRS cultures were collected 8, 24, and 48 h, corresponding to the exponential, early stationary, and late stationary phases, respectively. At these time-points, both the extracellular and the cell-associated activity were investigated. The supernatants were always incapable of hydrolyzing hesperidin (<2.0%), without any statistically significant difference during the course of the fermentation (*p* > 0.05). On the contrary, all the extracts were capable of hydrolyzing hesperidin, confirming that the activity is intracellular. Hydrolysis by cell extracts was highest during the exponential phase (*p* < 0.05), albeit the extracts from the early and late stationary phases were still active in hydrolyzing hesperetin (Figure 4).

## 4. Discussion

This study explored the role of gut microbiota and bifidobacteria in the release of the aglycones from two major rutinosides, hesperidin and rutin, whose corresponding aglycones hesperetin and quercetin present antioxidant, anti-inflammatory, and exert protective effects against cancer and cardiovascular diseases [2,3,4].

Rutin and hesperidin were subjected *in vitro* to the action of resting cells fecal microbiota. The ability of the microbiota to remove at some extent the rutinose moiety during 24 h of incubation was evident in all the samples. However, despite biotransformations being performed with very dense bacterial communities (approx. 10^10^ cfu/mL, resulting from 1:50 dilution of feces), the complete hydrolysis of rutinosides was never obtained. On the other hand, complete hydrolysis of chlorogenic acid was obtained in a similar study, in which the phytochemical was incubated with fecal microbiota under the same experimental conditions [22]. The disappearance of hesperidin was associated to a stoichiometric production of hesperetin, with yields ranging between 34% and 53%. Hydrolysis yields are likely affected by the low water solubility of hesperidin. Furthermore, the fact that the transformation of hesperetin did not proceed further, indicates that it is a somehow recalcitrant molecule.

The release of quercetin from rutin was successful in all the samples, with aglycone yields ranging from 15% to 58%. In two cases, the sum of quercetin and residual rutin was very low on molar basis, with a limited amount of quercetin accumulated in face of the nearly complete disappearance of rutin. This evidence can be due to the unlikely removal of the sole rhamnose moiety, or to transformations taking place at the expense of the aglycone. Consistently with this latter hypothesis, a previous study demonstrated that quercetin is transient in rutin-supplemented fecal cultures, being rapidly subjected to ring fission and transformation into hydroxyphenylacetic acid derivatives [25].

Bifidobacteria are important members of the saccharolytic population of intestinal bacteria, being able to produce several diverse glycosyl-hydrolases that are necessary to hydrolyze a number of oligo- and polysaccharides into fermentable sugars [18,26]. Abundant genomic data of sequenced bifidobacteria confirm the remarkable array of genes encoding for glycosyl hydrolases, whose involvement in the release of bioactive aglycones from dietary polyphenols has recently been claimed. However, the real contribution of *Bifidobacterium* species in the hydrolysis of specific polyphenols still needs to be proven.

Among the six tested species, mostly *B. catenulatum* and *B. pseudocatenultum* were able to hydrolyze hesperidin, by means of a cell-associated hydrolytic activity. The strains that were active in culture also presented active cell extracts, while the strains that were inactive in culture gave cell extracts that did not yield hesperetin. Therefore, among the strains herein tested, the inability of hydrolyzing hesperidin was not due to the lack of a suitable transport system. In agreement with the behavior of other glyco-conjugated polyphenols such as lignans and soy isoflavones, all the bifidobacteria were incapable of carrying out any further biotransformation of the aglycone [20,21]. In fact, for the strains able to hydrolyze hesperidin, the amount of hesperidin consumed corresponded to the amount of hesperetin yielded. Unlike hesperidin, rutin was utterly resistant to hydrolysis by bifidobacteria. The absence of hydrolytic activity in both supernatants and cell extracts established the lack of any glycosyl-hydrolase activity able to carry out rutin hydrolysis.

A previous study by Aura *et al.*, investigating the deglycosylation of cyanidin glycosides, demonstrated that rutinose removal from rutinosides requires the intervention of a α-rhamnosidase initially, and then of β-glycosidase [27]. The former activity represents the rate limiting step, due to the lower levels of α-rhamnosidase within the gut microbiota, while the latter proceeds much more rapidly, β-glucosidase being much more abundant [27].

An α-l-rhamnosidase capable of hydrolyzing diverse rhamnoglycoside-conjugated phytochemicals was recently cloned from *Bifidobacterium dentium* (GenBank: AGS77942.1) by Bang *et al.* [28]. A homologue of this gene can be found in the genome of *B. pseudocatenulatum* IPLA36007 (GenBank: KEF28006.1) [29], whose protein sequence exhibits an identity of 78.99% with the homologue of *B. dentium*. A TBLASTN search revealed that most of the available genome sequences of bifidobacteria, aside from *B. pseudocatenulatum* and *B. dentium,* do not bear homologues of KEF28006.1 or AGS77942.1, the sole exceptions being *B. kashiwanohense* PV20-2 and *B. breve* JCM 7019. It is interesting that 16 genome sequences are currently available for strains belonging to *B. breve* and that only one bears a gene encoding a putative α-l-rhamnosidase, homologue to those of *B. pseudocatenulatum* and *B. dentium*. This observation agrees with the result that only one out of six strains of *B. breve* hydrolyzed hesperidin in the present screening.

## 5. Conclusions

The data herein presented demonstrated that *B. pseudocatenulatum* can hydrolyze certain rutinose-conjugated polyphenols, such as hesperidin, thus releasing the aglycone form. Therefore, *B. pseudocatenulatum* may potentially contribute to the bioavailability of this class of molecules. However it remains to be clarified whether this species may exert a role in the colonic ecosystem to such an extent to effectively improve colonic absorption *in vivo*.
